# Faster Visual Information Processing in Video Gamers Is Associated With EEG Alpha Amplitude Modulation

**DOI:** 10.3389/fpsyg.2020.599788

**Published:** 2020-12-08

**Authors:** Yannik Hilla, Jörg von Mankowski, Julia Föcker, Paul Sauseng

**Affiliations:** ^1^Research Unit of Biological Psychology, Department of Psychology, Ludwig-Maximilians-Universität München, Munich, Germany; ^2^Chair of Communication Networks, Technische Universität München, Munich, Germany; ^3^School of Psychology, College of Social Sciences, University of Lincoln, Lincoln, United Kingdom

**Keywords:** theory of visual attention (TVA), attentional control, short-term memory, learning to learn, cognitive improvement in video gamers, knowledge system

## Abstract

Video gaming, specifically action video gaming, seems to improve a range of cognitive functions. The basis for these improvements may be attentional control in conjunction with reward-related learning to amplify the execution of goal-relevant actions while suppressing goal-irrelevant actions. Given that EEG alpha power reflects inhibitory processing, a core component of attentional control, it might represent the electrophysiological substrate of cognitive improvement in video gaming. The aim of this study was to test whether non-video gamers (NVGs), non-action video gamers (NAVGs) and action video gamers (AVGs) exhibit differences in EEG alpha power, and whether this might account for differences in visual information processing as operationalized by the theory of visual attention (TVA). Forty male volunteers performed a visual short-term memory paradigm where they memorized shape stimuli depicted on circular stimulus displays at six different exposure durations while their EEGs were recorded. Accuracy data was analyzed using TVA-algorithms. There was a positive correlation between the extent of post-stimulus EEG alpha power attenuation (10–12 Hz) and speed of information processing across all participants. Moreover, both EEG alpha power attenuation and speed of information processing were modulated by an interaction between group affiliation and time on task, indicating that video gamers showed larger EEG alpha power attenuations and faster information processing over time than NVGs – with AVGs displaying the largest increase. An additional regression analysis affirmed this observation. From this we concluded that EEG alpha power might be a promising neural substrate for explaining cognitive improvement in video gaming.

## Introduction

There is convincing evidence that playing commercially available video games, in particular action video games, such as Battlefield V (EA DICE; Stockholm), may improve cognitive functions – ranging from perception (Dye et al., 2009; Li et al., 2009, 2010; Bejjanki et al., 2014), over memory (Blacker and Curby, 2013; Blacker et al., 2014; McDermott et al., 2014; Pavan et al., 2019), probabilistic inference (Green et al., 2010; Schenk et al., 2017), and executive control (Colzato et al., 2010; Cain et al., 2012; Green et al., 2012; Strobach et al., 2012) to attentional deployment (Greenfield et al., 1994; Green and Bavelier, 2003; Chisholm and Kingstone, 2012; Cain et al., 2014; Wu and Spence, 2013). Thus, video gaming might represent a promising tool both for investigating human learning and therapeutic use in clinical populations (e.g., in patients with amblyopia, see Gambacorta et al., 2018). In this sense, for instance, EndeavorRx^TM^ (Akili Interactive Labs, Boston, MA, United States), a racing video game customized to treat children with ADHD (Kollins et al., 2020), was approved by the Food and Drug Administration (FDA), recently.

However, in order to apply commercially available video games (e.g., Battlefield V) for such purposes, the mechanisms underlying their effects need to be understood more in detail: one prominent attempt to explain how in particular action video games may improve cognitive processing is the idea that they affect one specific cognitive domain which several cognitive functions have in common – also known as learning to learn approach (Bavelier et al., 2012b; Green and Bavelier, 2012). According to this approach (Bavelier et al., 2012b; Green and Bavelier, 2012), playing action video games may improve video gamers’ probabilistic inference, which may enhance additional cognitive processes that rely on probabilistic inference, e.g., perception (Deroy et al., 2016) and attention (Rao, 2005). Thus, action video gamers (AVGs) might not outperform non-video gamers (NVGs) in a paradigm right from the start but after a time course of learning. Recently this approach has been developed further as a significant body of research indicated that foremost cognitive functions related to top–down attentional deployment were affected by video gaming (Bediou et al., 2018; Bavelier and Green, 2019). Hereby, Bavelier and Green (2019) suggested that the conjunction between reward-related learning and attentional control might be a core mechanism underlying improvements in cognitive processing related to video gaming. The idea here was that video gaming might train video gamers to select goal-relevant actions over goal-irrelevant actions which, in turn, might lead to more efficient cognitive processing. The basis for this was supposed to be an increase in dopaminergic transmission as video games seem to elicit learning mechanisms similar to operant conditioning, and an enhanced suppression of irrelevant information processing while relevant information processing may be facilitated – which is considered as attentional control in this context (Bavelier and Green, 2019).

In support of this, Koepp et al. (1998) showed that dopaminergic transmission of the left striatum increased during action video gaming; and Kühn et al. (2011) found that adolescents who frequently played video games exhibited larger gray matter volumes and stronger blood oxygenation level dependent signals in the left ventral striatum compared to adolescents with irregular gaming behavior while performing the Monetary Incentive Delay Task. Furthermore, Tanaka et al. (2013) elaborated that AVGs showed larger gray matter volumes in the right posterior parietal cortex than NVGs, and Bavelier et al. (2012a) as well as Föcker et al. (2018) demonstrated that AVGs exhibited different blood oxygenation level dependent signals in brain areas of a dorsal fronto-parietal top–down attention network (Corbetta et al., 2008) compared to NVGs, while performing attention demanding paradigms. Moreover, AVGs were shown to suppress distractors more efficiently than NVGs and NAVGs as reflected by stronger modulations of steady-state visual evoked potentials in the electroencephalogram (EEG) (Mishra et al., 2011; Krishnan et al., 2013); and, they showed stronger attention related amplitude responses in event-related potentials in the EEG compared to NVGs, for instance, in the anterior N1, P2, and P3 (Wu et al., 2012; Föcker et al., 2019).

However, these neural signatures were mostly (if at all) associated with modulations of attentional deployment, but rarely with modulations in additional cognitive functions (but see also, Tanaka et al., 2013). Thus, these data do not really allow any conclusions as to whether inter-individual differences in these neural substrates may also functionally apply to other cognitive processes. One promising candidate to fill this gap and relate inter-individual differences in neural signatures of attentional control with modulations in additional cognitive functions may be brain oscillatory activity in the frequency range from 8 to 14 Hz – also known as alpha oscillations. According to the inhibition-timing hypothesis (Klimesch et al., 2007b), amplitude modulations of EEG alpha oscillations represent a neural substrate of top–down inhibitory processing (for a validation of and new vistas on the inhibition-timing hypothesis, see also Jensen and Mazaheri, 2010; Fries, 2015). More specifically, the inhibition-timing hypothesis suggests that an increase in EEG alpha power after processing sensory information [also known as event-related synchronization (ERS)] goes along with an increase in inhibitory processing; while a decrease in EEG alpha power after processing sensory information [also known as event-related desynchronization (ERD)] may go along with disinhibition (see also Pfurtscheller and Lopes da Silva, 1999). Inhibitory processing, in turn, is a core property of attention (Chun et al., 2011). Therefore, modulations in EEG alpha power are assumed to represent a neural substrate of attentional control (Klimesch, 2012). Crucially, such modulations were shown to play a considerable role in cognitive functions associated with attentional control – for instance, in perception (Klimesch et al., 2011), (visual short-term) memory (Sauseng et al., 2009; Nenert et al., 2012), executive control (Sauseng et al., 2006), and probabilistic inference (Spaak et al., 2016). Hence EEG alpha power modulations represent a promising candidate for investigating neural mechanism of cognitive improvement related to video gaming. However, in this regard, it needs to be considered that alpha oscillatory activity is highly task-dependent (Klimesch et al., 2011): for instance, while EEG alpha power seems to decrease in anticipation of the time point (Nobre and van Ede, 2018), spatial position (Spaak et al., 2016) or identity (Capotosto et al., 2009) of an up-coming target in visual detection paradigms; it tends to increase in anticipation of stimuli in visual short-term memory paradigms and decrease after stimulus processing later on (e.g., Nenert et al., 2012). Moreover, in line with the inhibition-timing hypothesis, alpha activity tends toward increasing in response to distractors (Worden et al., 2000; Sauseng et al., 2009). Furthermore, EEG alpha oscillations can be sub-divided into lower alpha frequency bands (e.g., from 6.42 to 9.75 Hz, see Freunberger et al., 2008) that seem to be related merely to attentional processing, and medium or upper alpha frequency bands (e.g., from 9.17 to 13 Hz, see Freunberger et al., 2008) that appear to be correlated with higher level cognitive processing, e.g., when attention is deployed in concert with perception and memory (Klimesch, 1997, 2012; Klimesch et al., 2011). But, the frequency range of lower, medium and upper alpha bands may drastically vary depending on data recording and analysis protocols.

Hazarika et al. (2018) and Hazarika and Dasgupta (2020) have already provided some evidence that AVGs might exhibit differences in EEG alpha oscillatory activity as they found that AVGs showed larger relative EEG alpha power values than NVGs while performing a Corsi block-tapping task and the Bivalent Shape Task. But their results should be considered with caution as their procedures to estimate participant’s relative EEG alpha power values was problematic. One of the main issues here may be that each participant’s relative EEG alpha power value was estimated based on trials of different lengths, while performing the Corsi block-tapping task since the length of a trial depended on the individual performance of a participant (Hazarika and Dasgupta, 2020). Thus, the estimates are hardly comparable between subjects and groups. On top of that, it appeared that Hazarika et al. (2018) and Hazarika and Dasgupta (2020) did not control for potential contaminations by stimulus exposure duration, time on task or task requirements, though EEG alpha power was shown to decay over time, to be modulated by time on task and to be highly task-specific (Klimesch et al., 2011; Benwell et al., 2019). Furthermore, they reported relative EEG alpha power estimates that were quite broad (7.8–15.6 Hz). Thus, the estimates might reflect the impact of not only alpha oscillatory activity but also other frequency bands. In conclusion, further research on the functional role of EEG alpha oscillatory activity in inter-individual differences in cognitive processing between video gamers and control participants is required to understand neural mechanisms underlying cognitive improvements due to video gaming.

In this regard, the aim of this study was to investigate whether habitual gaming behavior might modulate EEG alpha power while processing stimuli in a visual-short-term memory paradigm, and whether such modulations might be relatable to inter-individual differences in visual attentional information processing. For this, we applied a computational modeling approach based on the theory of visual attention (TVA; Bundesen, 1990; Bundesen et al., 2015) because it allows the computation of several parameter values associated with visual attentional processing, such as speed of information processing (C) or the maximum capacity of the visual short-term memory store (K) (Kyllingsbaek, 2006; Dyrholm et al., 2011). For this, participants’ accuracy data are fit to exponential graphs using TVA-algorithms to determine their TVA C and K parameter values based on the assumptions (1) that the capacity of the visual short-term memory store is limited, (2) but that all visual information is processed in parallel and (3) hence needs to be filtered according to subjectively relevant criteria using attentional deployment. Hereby, the y-asymptotic levels of the exponential graphs represent K parameter values and slope lines that intersect the exponential graph on the *x*-axis C parameter values. We decided on investigating TVA C and K parameter values in particular since they were likely related with EEG alpha activity as they have been associated with the posterior N1 and the contralateral delay activity (CDA), respectively (Wiegand et al., 2014b) – two event-related potentials that have been discussed to be linked to EEG alpha activity (Klimesch et al., 2004; Gruber et al., 2005; Klimesch et al., 2007a; van Dijk et al., 2010); and because ERD in the medium or upper alpha frequency band is considered a crucial neural signature in visual short-term memory (Klimesch, 1997, 2012; Sauseng et al., 2009; Nenert et al., 2012). An additional reason was that video gamers were shown to exhibit larger TVA C parameter values than NVGs (Wilms et al., 2013) – an effect already replicated (Schubert et al., 2015). Hence, the effect was considered suitable to investigate whether inter-individual differences in event-related EEG alpha power modulations between video gamers and NVGs might predict differences in TVA C parameter values – but not in TVA K parameter values since video gamers and NVGs did not seem to differ in the capacity of visual short-term memory as operationalized by the TVA K parameter (Wilms et al., 2013; Schubert et al., 2015).

Therefore, we hypothesized (1) that the extent of participants’ ERD in the medium or upper alpha frequency band after processing stimuli presented in a visual short-term memory paradigm may be correlated with TVA C and K parameter values, respectively; (2) that habitual video gaming might modulate EEG alpha power as video gaming seems to impact on attentional control; and (3) that inter-individual differences in ERD in the medium or upper alpha frequency bands between video gamers and non-video gamers might go along with inter-individual differences in TVA C parameter values. On top of that, we expected (4) that different lengths of exposure durations used to compute TVA parameter values and time on task as operationalized by experimental blocks in our paradigm might contaminate our EEG alpha power estimates – which is why we controlled for these factors in our statistical analyses (Klimesch et al., 2011; Benwell et al., 2019).

## Materials and Methods

### Participants

We recruited participants via flyers published on mailing lists and internet platforms, stating that we were looking for male volunteers to participate in an EEG study on perceptual processing. Thus, we did not explicitly recruit video gamers, but we used a cover story to prevent selection bias and expectation effects from confounding the data (Boot et al., 2011; Schubert and Strobach, 2012).

A screening was conducted to control whether prospective volunteers were suitable for participating in the study. Only male individuals between 18 and 40 years of age with normal or corrected-to-normal vision and no history of neurological or psychiatric disorders were eligible. We recruited only male participants as their representation among video gamers was likely larger than that of females (Entertainment Software Association, 2019) – a procedure which is quite common in gaming research (e.g., Green and Bavelier, 2007). In total 40 healthy male German- or English-speaking volunteers with normal or corrected-to-normal vision participated in our study. Fifteen were classified as NVGs, 15 as NAVGs and 10 as AVGs. The data of two participants were excluded due to poor EEG data quality. Thus, data of 14 NVGs (*M*_age_ = 24.93 years, age range = 22–30 years), 15 NAVGs (*M_age_* = 22.73 years, age range = 19–32 years) and 9 AVGs (*M_age_* = 24.89 years, age range = 21–31 years) were used for statistical analyses. Our sample size was comparable to sample sizes reported in studies that followed a similar methodological approach (Wilms et al., 2013; Schubert et al., 2015), and in studies investigating other gaming effects (Li et al., 2009, 2011; Wu et al., 2012). The three groups did not differ in mean age, *F*(2,35) = 2.43, *p* = 0.103, and were similar in educational status (High School graduation vs. Bachelor’s Degree vs. Master’s Degree vs. German Diploma, Fisher’s exact test, *p* = 0.128). The study was approved by the local ethics review board. All volunteers gave written informed consent in accordance with the Declaration of Helsinki before their participation, and they were compensated with 3 Euros for participating in the screening and 10 Euros per hour spent on the EEG study, or participants received student lab tokens.

### Procedures and Materials

#### Video Gaming Questionnaire

Prior to testing, we asked participants to provide the names of a maximum of 10 video games they had played regularly and most often in the previous 12–24 months. In addition to that, we asked how many hours per week they had played the respective games on average in the previous 12–24 months. Each video game’s genre was determined based on the producers’ description. With regard to the classification scheme of Green et al. (2017), subjects were, then, classified as NVGs if in the previous 12–24 months, they had played first/third person shooter, action role play/adventure, sports/driving, real-time strategy/multi-player online platform video games each for a maximum of 1 h and non-action turn-based role play/fantasy, turn-based strategy/life simulation/puzzle, music or other video games each for a maximum of 3 h, but in total not more than 5 h per week on average. Participants were classified as AVGs if in the previous 12–24 months, they had played first/third person shooter, action role play/adventure games for at least 5 h per week on average. All remaining individuals were classified as NAVGs.

#### Visual Short-Term Memory Paradigm

Our paradigm was run on a computer with an AMD Athlon^TM^ II X2 B24 processor (AMD, Sunnyvale, CA, United States) and a 64-Bit Windows 7 operating system (Microsoft, Redmond, WA, United States). The paradigm was developed using *Python*’s (Version 3.7.3; Python Software Foundation) *Tkinter* library^1^ and run in *Spyder* (Version 3.3.3^2^). Stimuli were presented on a 17″/43 cm monitor (Acer Group, Taiwan) with a refresh rate of 60 Hz. Triggers were sent to the EEG computer using the *dportio.dll in-script plug-in*.^3^ Responses were given on a regular keyboard (KB212-B; Dell Technologies Inc., Round Rock. United States).

We developed our own TVA paradigm based on Vangkilde et al. (2011) with shape stimuli instead of letter stimuli to control for language confounds. Participants sat in a comfortable chair, approximately 80 cm from the screen, in a dimly lit room. Each trial started with the presentation of a gray blank screen. After 1002 ms, a white fixation cross was depicted in the center of the screen (0.72° × 0.72° of visual angle). After an additional 1002 ms, a stimulus display comprising 6 out of 10 white geometrical shapes (circle, ellipse, hexagon, diamond, pentagon, rhombus, square, star, trapezoid, triangle) was presented. Those shapes were displayed on an invisible circle at 30°, 90°, 150°, 210°, 270° and 330° around the fixation cross. The radius of the circle was 2.72° of visual angle. Each shape was located within an area of 2.29° × 2.29° of visual angle and could reach a maximum size of 2.08° × 2.08° of visual angle (e.g., square). Stimulus displays were presented at one of six different exposure durations (16.7, 33.4, 50.1, 83.5, 150.3, and 200.4 ms). Directly after the stimulus display, a mask display consisting of white squares of 2.29° × 2.29° of visual angle with random black polygons based on 8 random points was presented to interrupt processing of stimulus displays exactly at the end of the exposure duration. This mask display lasted for 501 ms. Thereafter, an instruction written in white letters was displayed in the center of the screen. Participants were asked to retrieve as many shapes as possible and to indicate which ones had been presented by pressing corresponding buttons on the keyboard (D: circle, F: ellipse, G: hexagon, H: diamond, J: pentagon, K: rhombus, C: square, V: star, B: trapezoid, N: triangle). Each key was marked with a glow-in-the-dark sticker of the corresponding shape. Participants were asked not to guess. Every new trial was initiated by pressing the space bar. Participants started the paradigm with a training block consisting of 24 trials. During this training, participants received feedback. If at least one response was incorrect within a trial, a black “X” covering 0.93° × 0.93° of visual angle was presented in the center of the screen for 501 ms. There were 210 stimulus displays, see equation (1) for details:

(1) $$\frac{10!}{\left(10-6\right)!*6!}=210$$

**FIGURE 1:**
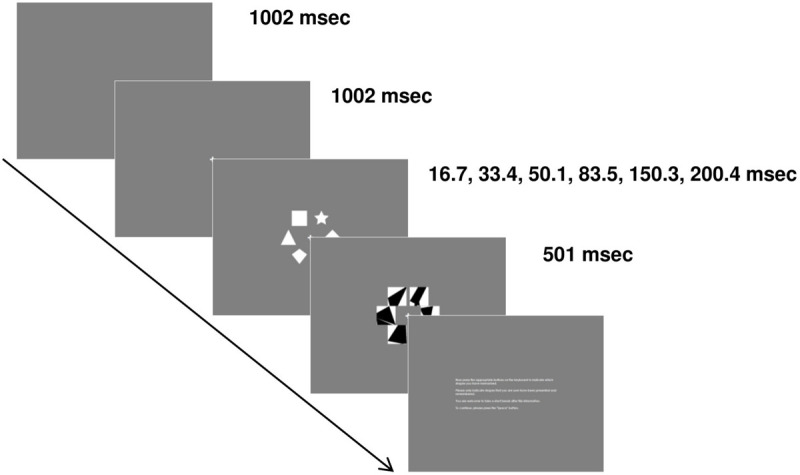
Paradigm. Participants performed a visual short-term memory paradigm where they had to memorize white shape stimuli depicted on an invisible circle in the center of the screen at six different exposure durations. Mask displays were used to prevent further processing. At the end of each trial, participants indicated which shapes they could memorize by pressing corresponding buttons on a keyboard.

Each stimulus display was presented once in an experimental block and 24 out of those 210 stimulus displays were randomly chosen for the training block. Thus, each participant performed one training block and two experimental blocks, which makes a total of 444 trials. During the training, each exposure duration was used four times and during each experimental block, each exposure duration was used 35 times. The sequence of trials was always random. The association between a stimulus display and an exposure duration was random. Participants were allowed to take a break between blocks and after each trial. For a scheme of an exemplary trial, see Figure 1.

#### Computation of TVA C and K Parameter Values

Estimating TVA C and K parameter values requires the manipulation of the exposure duration and the application of masks. Manipulating the exposure duration is necessary because TVA-algorithms estimate each participant’s TVA C and K parameter value by fitting their accuracy data at each exposure duration to an exponential distribution by using maximum-likelihood method. Hereby, the slope line that intersects the exponential graph on the *x*-axis reflects the value of the C parameter, and the *y*-asymptotic level of the exponential graph reflects the K parameter value (Bundesen, 1990; Kyllingsbaek, 2006; Dyrholm et al., 2011; Bundesen et al., 2015). The application of masks was necessary to prevent visual aftereffects that may otherwise have influenced stimulus processing (see Kyllingsbaek, 2006). As the exposure durations were such an essential element in the computation of TVA C and K parameter values, we inspected the temporal dynamics of the stimuli by using the *default_timer()* function of the *timeit* library^4^. Specifically, we recorded the time points when the in-script commands to visualize stimuli (fixation cross, stimulus displays, mask displays and instruction texts) were given (with a resolution of at least 1 ms) and computed the time differences between them. This, however, revealed that there had been minor imprecisions in the presentation times of the stimuli. Such imprecisions are unfortunately inevitable when using non-real time operating systems and working with a refresh-rate of 60 Hz. We corrected for those flaws by adding the mode value (rounded to the first decimal place) of each exposure duration’s deviation distribution to the expected exposure duration value. Thus, we changed the original exposure durations from 16.7, 33.4, 50.1, 83.5, 150.3, and 200.4 ms to 17.8, 35.6, 53.4, 89, 144.6, and 198 ms, respectively. We decided to add the mode value because it represents the most common realization of a distribution and therefore, arguably covers the most frequent exposure durations participants were confronted with while performing the paradigm. We then estimated each participant’s TVA C and K parameter value based on their accuracy data of the experimental trials by using the *LIBTVA toolbox* (Dyrholm et al., 2011) run in Matlab R2015a (Math Works, Natick, MA, United States). Finke et al. (2005) established that a minimum of 192 trials in a visual short-term memory paradigm would be sufficient to reliably estimate TVA C and K parameter values in 35 participants. Our visual short-term memory paradigm was comparable to the experimental design used in Finke et al.’s (2005) study and our participants performed 420 experimental trials. Therefore, we were confident that our TVA C and K parameter estimates were reliable.

#### EEG Data Recording

EEG recordings were stored on a computer with an AMD Athlon^TM^ 64 × 2 Dual Core Processor 5000 + processor (AMD, Sunnyvale, CA, United States) and a 64-Bit Windows 7 operating system (Microsoft, Redmond, WA, United States). EEGs were recorded by using a 64-channel BrainAmp DC amplifier and *Brain Vision Recorder Software* (Brain Products GmbH, Gilching, Germany). Sixty two Ag/AgCl electrodes mounted in a cap (EASYCAP, Herrsching, Germany) were arranged according to the 10–10 international EEG system. During recording, EEGs were referenced against the tip of the nose. Vertical and horizontal EOGs were mounted above the left eyebrow and on the left canthus of the left eye, respectively. EEGs were digitized with a sampling rate of 1000 Hz and filtered with a band-pass filter between 0.016 and 250 Hz. Impedances were kept below 10 kOhm.

#### Computation of EEG Alpha Power

EEG data were analyzed using *Brain Vision Analyzer 2* (BVA, Brain Products GmbH, Gilching, Germany). At first, raw data were inspected for large scale artifacts such as movement artifacts and artifacts caused by cable movements. Those artifacts were then manually excluded from further analysis using BVA’s *Raw Data Inspection tool*. Subsequently, a bandpass filter between 0.1 and 120 Hz and a notch filter of 50 Hz were applied to reduce slow voltage drifts, muscle artifacts and line noise. If necessary, bad channels were interpolated using the *Topographic Interpolation tool*. Afterward, the EEG was re-referenced to averaged common reference. Then, an *Ocular Correction ICA* was applied using the *Infomax Restricted Algorithm* to correct eye movement artifacts, such as blinks and saccades. After that, a second data inspection was conducted to exclude remaining muscle artifacts, uncorrected EOG artifacts and unspecific spikes. Data were resampled to 1024 Hz to prepare the data for an upcoming *Fast Fourier Transformation* with a resolution of 2 Hz. Data were segmented into segments of 500 ms before the presentation of stimulus displays and after the presentation of the mask display. Subsequently, Fast Fourier Transformation was applied to segments. Trials were then averaged into power spectra for each time window (pre-stimulus and post-mask) and each participant, separately. Then, EEG power ratios were computed by dividing average power spectra before stimulus presentation by average power spectra after mask presentation, see equation (2):

(2) $$E\,E\,G\,p\,o\,w\,e\,r\,r\,a\,t\,i\,o=\frac{a\,v\,e\,r\,a\,g\,e\,E\,E\,G\,P\,o\,w\,e\,r\,S\,p\,e\,c\,t\,r\,a_{p\,r\,e-s\,t\,i\,m\,u\,l\,u\,s}}{a\,v\,e\,r\,a\,g\,e\,E\,E\,G\,P\,o\,w\,e\,r\,S\,p\,e\,c\,t\,r\,a_{p\,o\,s\,t-m\,a\,s\,k}}$$

Afterward, we achieved *medium and upper alpha ratios* by exporting ratios averaged over occipital, parietal and occipitoparietal channels (O1, O2, Oz, P1, P2, P3, P4, P5, P6, P7, P8, PO3, PO4, PO7, PO8, POz, Pz) of frequency bands ranging from 10–12 and 12–14 Hz. At last, we log-transformed these medium and upper alpha ratios using log_10_ to prepare the data for statistical analyses based on general linear models. Ratio values > 0 indicated ERD, ratio values < 0 ERS.

We did not include segments ranging from the onset of stimulus displays to the onset of mask displays since we assumed that differences in the exposure durations might contaminate the EEG as they cause different on- and offset evoked potentials. In previous research, this issue was circumvented by combining data of two paradigms – one where masks had been applied but no EEG was recorded and one where masks were not applied but EEGs were recorded (Wiegand et al., 2014a,b, 2016). This procedure certainly allowed for a valid estimation of both TVA parameter values and event-related potentials. However, the shortcoming here may be that an association between the two components might be problematic because of both the temporal distinct recording times and the different task requirements. In contrast, to circumvent this issue, but with the shortcoming of having to neglect data ranging from the presentation of the stimulus display to the presentation of the mask display, we compared alpha power before stimulus processing with alpha power after mask processing. Note also that Hanslmayr et al. (2009) showed that estimates of alpha power modulations were quite stable at the rate of 15 trials and more using a simulation approach (see supplements of Hanslmayr et al., 2009). We used 35 trials to compute alpha power ratios for each exposure duration per block. Therefore, we were confident that our alpha ratios were reliable.

#### Statistical Analyses

We used *R* (R Core Team, 2018) to conduct statistical analyses and visualize results. Specifically, we used the *apaTables package* to generate tables (Standley, 2018); the *BayesFactor package* to compute Bayes factors (Moray and Rouder, 2018); the *cowplot*, the *ggplot2*, and the *RColorBrewer packages* to visualize data with colorblind-friendly color palettes (Neuwirth, 2014; Wickham, 2016; Wilke, 2019); the *dplyr package* (Wickham et al., 2019) to process data; the *ez package* to compute variance analytical procedures (Lawrence, 2016); the *performance package* to control model assumptions of regression and correlation models (Lüdecke et al., 2020); the *rstatix package* to control model assumptions of variance analytical procedures (Kassambara, 2020); and the *stats package* to conduct Pearson’s moment correlation tests. For variance analytical methods with between-subjects factors, we used *Shapiro–Wilk test* (Shapiro and Wilk, 1965) and *Levene’s test* (Levene, 1960) to control for assumptions of normality and variance homogeneity, respectively. In case of violations of normality, we, nevertheless, computed variance analytical methods since they were shown to be robust against such violations (Schmider et al., 2010). The assumption of homogeneity was met in all variance analytical models with between-subjects factors. For variance analytical methods with an additional within-subject factor, Mauchly’s tests were used to test the assumption of sphericity. In case of a violation of the assumption of sphericity, the *Greenhouse–Geisser correction* was applied (Abdi, 2010). In general, we computed variance analytical procedures based on type II sums of squares since our group sample sizes were unbalanced (Langsrud, 2003). To quantify significant variance analytical results, we reported *generalized eta squared* ($\eta_{\text{G}}^{2}$; Olejnik and Algina, 2003). For regression and correlation models, we first screened for outliers using *Cook’s distance* (Cook, 1977), then the assumptions of normality and of homoscedasticity were controlled. All our regression and correlation models met the assumptions of normality and homoscedasticity. To quantify the strengths of significant associations, we reported Pearson’s correlation coefficient *r*; and to quantify model fits of regression models, we reported the determination coefficient *R*^2^. Besides, we computed and reported Bayes factors. These indicate how likely the data occur either under the assumption of H_1_ or H_0_. For instance, a Bayes factor *BF*_10_ = 4 indicates the data are four times more in support of the H_1_ than the H_0_. In contrast, a Bayes factor *BF*_01_ = 3 indicates the data are three times more in support of the H_0_ than the H_1_. We only computed Bayes factors for significant results. We corrected for multiple comparisons using *Bonferroni method* (Bland and Altman, 1995).

At first, we computed 4 one-tailed Pearson’s moment correlation tests with average medium or upper alpha ratios and TVA C or K parameter values as variables. We expected positive correlation coefficients as we hypothesized that each participant’s ERD in the medium or upper alpha frequency band may be correlated with their TVA C and K parameter values, respectively. For this, we had to average each participant’s medium and upper alpha ratio over each level of exposure duration across all blocks to achieve one average medium and upper alpha ratio that could be matched with each participant’s individual TVA C and K parameter value. Note, however, that this was problematic since EEG alpha power might decay over time (Klimesch et al., 2011) – thus, average medium or upper alpha ratios might be contaminated by different lengths of exposure durations; and, EEG alpha power is modulated by time on task (Benwell et al., 2019) – thus, average medium or upper alpha ratios might differ at least between blocks. Afterward, we computed two three-way ANOVAs with GROUP (NVGs vs. NAVGs vs. AVGs) as between-subjects factor, EXPOSURE DURATION (17.8, 35.6, 53.4, 89, 144.6, and 198 ms) and TIME ON TASK (Block 1 vs. Block 2) as within-subject factors and medium and upper alpha ratios as dependent variables, respectively. In doing so, we investigated the influence of the factor GROUP on medium and upper alpha ratios as we expected that video gamers exhibit different event-related EEG alpha power modulations than NVGs. In addition to that, we investigated the influence of EXPOSURE DURATION and TIME ON TASK on medium and upper alpha ratios to control for modulations by different lengths of exposure durations (Klimesch et al., 2011) and time on task (Benwell et al., 2019). Afterward, we conducted two two-way ANOVAs with GROUP (NVGs vs. NAVGs vs. AVGs) as between-subjects factor, TIME ON TASK (Block 1 vs. Block 2) as within-subject factor and TVA C and K parameter values as dependent variables, respectively. We expected inter-individual differences in ERD in medium or upper alpha frequency bands between video gamers and NVGs to go along with inter-individual differences in TVA C parameter values, but not in K parameter values. However, if this was the case because EEG alpha power modulations and TVA C and K parameter values were associated, TVA C and K parameter values may also be modulated by time on task – and probably by different lengths of exposure durations. Therefore, we had to estimate TVA C and K parameter values for each block separately by estimating each participant’s TVA C and K parameter value based on their 210 experimental trials of each block – which is still a sufficient trial number to estimate reliable values (see Finke et al., 2005). However, we could not control for potential influences of exposure durations since TVA C and K parameter values are estimated based on accuracy data of all exposure durations, and hence it is not possible to compute them for one individual exposure duration (see above).

Finally, we conducted regression analyses with each participant’s TVA parameter difference value as criterion variable [see equation (3)], each participant’s alpha ratio difference value as predictor variable [see equation (4)], and GROUP as dummy variable (NVGs vs. NAVGs vs. AVGs) where NVGs were used as reference to put the results of significant ANOVAs for alpha ratios and for TVA parameter values in relation to each other.

T⁢V⁢A⁢p⁢a⁢r⁢a⁢m⁢e⁢t⁢e⁢r⁢d⁢i⁢f⁢f⁢e⁢r⁢e⁢n⁢c⁢e⁢v⁢a⁢l⁢u⁢e

  =T⁢V⁢A⁢p⁢a⁢r⁢a⁢m⁢e⁢t⁢e⁢r⁢v⁢a⁢l⁢u⁢eB⁢l⁢o⁢c⁢k⁢ 2

(3)    -T⁢V⁢A⁢p⁢a⁢r⁢a⁢m⁢e⁢t⁢e⁢r⁢v⁢a⁢l⁢u⁢eB⁢l⁢o⁢c⁢k⁢ 1

a⁢l⁢p⁢h⁢a⁢r⁢a⁢t⁢i⁢o⁢d⁢i⁢f⁢f⁢e⁢r⁢e⁢n⁢c⁢e⁢v⁢a⁢l⁢u⁢e

  =a⁢v⁢e⁢r⁢a⁢g⁢e⁢a⁢l⁢p⁢h⁢a⁢r⁢a⁢t⁢i⁢oB⁢l⁢o⁢c⁢k⁢ 2

(4)    -a⁢v⁢e⁢r⁢a⁢g⁢e⁢a⁢l⁢p⁢h⁢a⁢r⁢a⁢t⁢i⁢oB⁢l⁢o⁢c⁢k⁢ 1

The regression model was built as described in equation (5), where *Y* indicates TVA parameter difference values, *X* alpha ratio difference values, *i* the index corresponding to a participant, α the intercept of the reference model (NVGs), β_*1*_ the slope of the reference model, *D*_ij_ the dummy variable of group *j*, β_*2*_ the difference in intercepts between NAVGs and NVGs, β_*3*_ the difference in intercepts between AVGs and NVGs, β_*4*_ the difference in slopes between NAVGs and NVGs, β_*5*_ the difference in slopes between AVGs and NVGs and ε_i_ the residual term. We tested the significance of the whole model but not of the individual β_j_ parameter values. This is because β_j_ values may be potentially biased because of small group sample sizes yielding significant results without enough statistical power.

$$Y_{i}=\alpha +\beta_{1}\,X_{i}+\beta_{2}\,D_{i\,N\,A\,V\,G\,s}+\beta_{3}\,D_{i\,A\,V\,G\,s}+\beta_{4}\,\left(X_{i}\,D_{i\,N\,A\,V\,G\,s}\right)$$

(5) $$\;+\beta_{5}\,\left(X_{i}\,D_{i\,A\,V\,G\,s}\right)+\varepsilon_{i}$$

## Results

**FIGURE 2:**
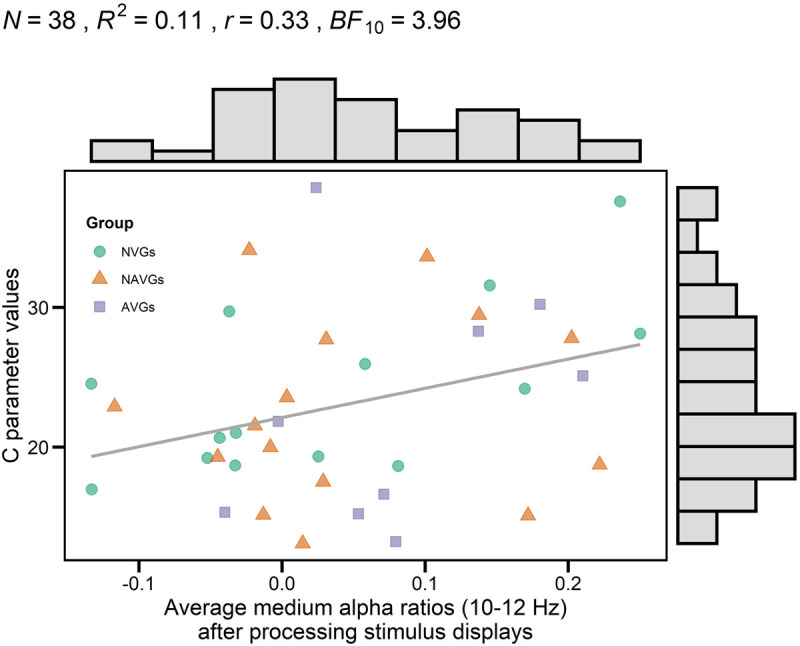
Correlation between theory of visual attention speed of information processing parameter values (C parameter values) and average medium alpha ratios (10–12 Hz) after processing stimulus displays. Averages were computed based on log-transformed medium alpha ratios using log_10_. Average alpha ratios > 0 indicate event-related desynchronization (ERD); average alpha ratios < 0 event-related synchronization (ERS). *N* indicates the number of participants, *R*^2^ the estimate of the determination coefficient, *r* the estimate of Pearson’s correlation coefficient and *BF*_10_ the evidence in support of the H_1_. Histograms opposite to the *y*- and *x*-axes indicate the distribution of C parameter values and average medium alpha ratios, respectively. Group affiliation is indicated by different colors (NVGs: green, NAVGs: orange, AVGs: purple) and shapes (NVGs: circle, NAVGs: triangle, AVGs: square). There was a marginally significant positive correlation between C parameter values and average medium alpha ratios, indicating that faster speed of information processing was associated with an increase in alpha power attenuation in a frequency range from 10 to 12 Hz after stimulus processing (i.e., ERD).

There was a marginally significant positive correlation between average medium alpha ratios and C parameter values, *R*^2^ = 0.11, *r* = 0.33, *t*(36) = 2.06, *p*_uncorrected_ = 0.023, *p*_corrected_ = 0.093, *BF*_10_ = 3.96. But none of the other correlations reached significance (for a more detailed description, see Table 1). This correlation indicates that an increase in alpha power attenuation in a frequency band from 10 to 12 Hz after processing stimulus displays (i.e., ERD) goes along with an increase in speed of information processing. For a visualization of the association, see Figure 2.

**TABLE 1:** One-tailed Pearson’s moment correlation tests with average medium or average upper alpha ratios and theory of visual attention speed of information processing parameter values (C parameter values) or visual short-term memory capacity parameter values (K parameter values) as variables.

**Model**	***r***	***R*^2^**	***df***	***t***	***p***	***p*_Bonferroni_**	***BF*_10_**
K parameter values and average medium alpha ratios	0.08	0.01	36	0.51	0.307	1.000	
K parameter values and average upper alpha ratios	0.20	0.04	36	1.23	0.114	0.454	
C parameter values and average medium alpha ratios	0.33	0.11	36	2.06	0.023	0.093	3.96
C parameter values and average upper alpha ratios	0.12	0.01	36	0.70	0.244	0.977	

**r* indicates Pearson’s correlation coefficients. *R*^2^ indicates determination coefficients. *df* indicates degrees of freedom. *p* indicates uncorrected *p*-values. *p*_*Bonferroni*_ indicates *p*-values corrected by means of Bonferroni method. *BF*_10_ indicates Bayes factors in favor of the H_1_. *BF*_10_ was only reported for (marginally) significant results after application of the Bonferroni method. Raw medium and upper alpha ratios were log-transformed using log_10_ to prepare them for statistical analyses based on linear models.*

**FIGURE 3:**
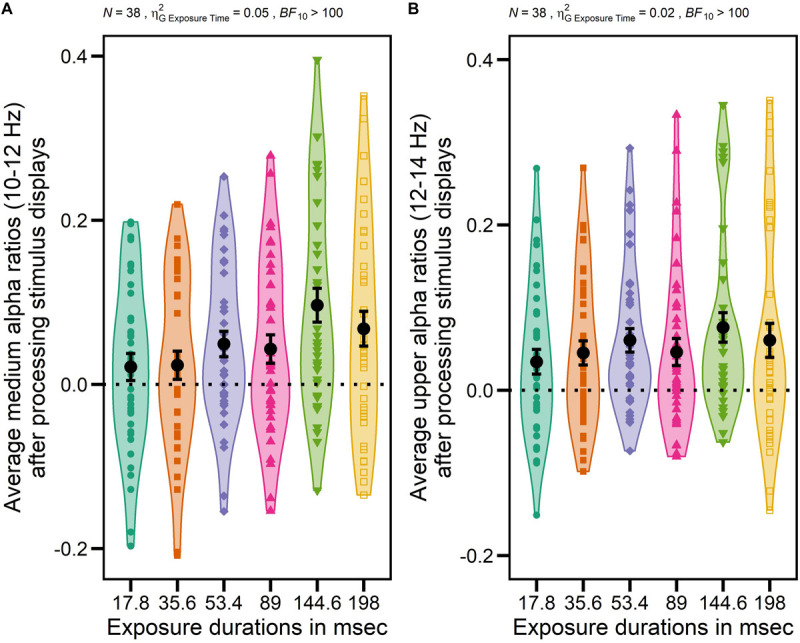
Impact of different exposure durations on average medium alpha ratios (10–12 Hz) and average upper alpha ratios (12–14 Hz) after processing stimulus displays. Averages were computed based on log-transformed medium and upper alpha ratios using log_10_. Average alpha ratios > 0 indicate event-related desynchronization (ERD); average alpha ratios < 0 event-related synchronization (ERS). *N* indicates the number of participants, $\eta_{G}^{2}$ generalized eta-squared, *BF*_10_ the evidence in support of the H_1_, and the dotted horizontal line indicates the absence of any modulation of alpha activity (alpha ratio = 0). Participants’ individual average medium or upper alpha ratios for each exposure duration are indicated by different colors (17.8 ms: green, 35.6 ms: orange, 53.4 ms: purple, 89 ms: pink, 144.6 ms: light green, 198 ms: yellow) and shapes (17.8 ms: circle, 35.6 ms: square, 53.4 ms: diamond, 89 ms: triangle, 144.6 ms: triangle upside down, 198 ms: empty square). Total averages over all participants of each exposure duration are indicated by black dots. Error bars indicate standard errors. The main effect EXPOSURE DURATION was significant for medium alpha ratios after processing stimulus displays indicating that the extent of alpha power attenuation in a frequency band from 10 to 12 Hz after stimulus processing increased in relation to increasing exposure durations up to 144.6 ms (i.e., ERD) but started to decrease with even longer exposure duration again (i.e., ERS) **(A)**. A similar effect was found for upper alpha ratios **(B)**.

However, as expected, the ANOVA with GROUP (NVGs vs. NAVGs vs. AVGs) as between-subjects factor, EXPOSURE DURATION (17.8, 35.6, 53.4, 89, 144.6, and 198 ms) and TIME ON TASK (Block 1 vs. Block 2) as within-subject factors and medium alpha ratios as dependent variable revealed a significant main effect EXPOSURE DURATION, *F*(3.72,130.37) = 16.80, *p* = 0.000, $\eta_{\text{G}}^{2}$ = 0.05, *BF*_10_ > 100, indicating that the extent of alpha power attenuation in the frequency band from 10 to 12 Hz after stimulus processing was differentially modulated by exposure durations. Specifically, the extent of attenuation appeared to increase in relation to increasing exposure durations up to 144.6 ms, and to decrease with even longer exposure durations (for a visualization see Figure 3A). Hence, the correlation between average medium alpha ratios and C parameter values might be slightly contaminated by differential effects of exposure durations as medium alpha ratios were averaged without considering these intra-individual differences. Nevertheless, this procedure was inevitable as alpha ratios needed to be matched to the individual TVA parameter values, respectively (see **Computation of TVA C and K parameter values** for a revision). A similar effect was found for upper alpha ratios, *F*(3.63,127.05) = 5.41, *p* = 0.001, $\eta_{\text{G}}^{2}$ = 0.02, *BF*_10_ > 100 (see Figure 3B for a visualization).

**FIGURE 4:**
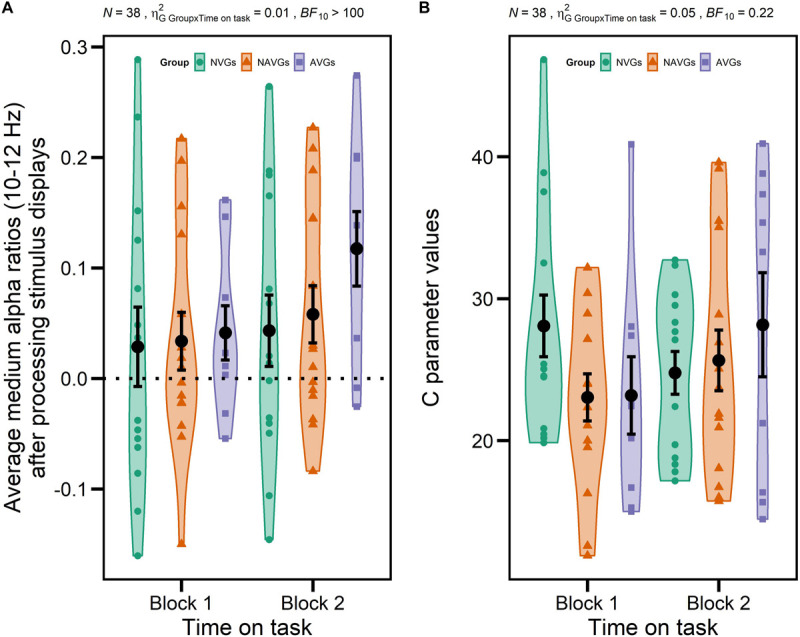
Significant interactions GROUP [non-video gamers (NVGs) vs. non-action video gamers (NAVGs) vs. action video gamers (AVGs)] × TIME ON TASK (Block 1 vs. Block 2) for average medium alpha ratios (10–12 Hz) after processing stimulus displays and theory of visual attention speed of information processing parameter values (C parameter values). Average medium alpha ratios were computed based on log-transformed medium alpha ratios using log_10_. Average alpha ratios > 0 indicate event-related desynchronization (ERD); average alpha ratios < 0 event-related synchronization (ERS). *N* indicates the number of participants, $\eta_{\text{G}}^{2}$ generalized eta-squared, *BF*_10_ the evidence in support of the H_1_, and the dotted horizontal line indicates the absence of any modulation of alpha activity (alpha ratio = 0). Group affiliation is indicated by different colors (NVGs: green, NAVGs: orange, AVGs: purple) and shapes (NVGs: circle, NAVGs: triangle, AVGs: square). Total averages over all participants of each level combination of GROUP and TIME ON TASK are indicated by black dots. Error bars indicate standard errors. The interaction for average medium alpha ratios indicates that participants in each group exhibited a larger alpha power attenuation in a frequency band from 10 to 12 Hz (i.e., ERD) in the second experimental block relative to the first one, but that AVGs showed the largest increase (in ERD) **(A)**. The interaction for C parameter values indicates that NVGs exhibited larger C parameter values than video gamers in the first experimental block, but that video gamers showed larger C parameter values in the second experimental block with AVGs displaying the largest increase **(B)**. Thus, the temporal dynamics of C parameter values were nicely paralleled by average medium alpha ratios in video gamers but not in NVGs.

But, more importantly, this ANOVA also revealed a significant interaction GROUP × TIME ON TASK for medium alpha ratios, *F*(2,35) = 4.80, *p* = 0.014, $\eta_{\text{G}}^{2}$ = 0.01, *BF*_10_ > 100, suggesting that there were differential intra-individual differences in alpha power attenuation in the frequency band from 10 to 12 Hz between experimental blocks between video gamers and control participants. Specifically, participants exhibited larger medium alpha ratios – i.e., a stronger ERD – in Block 2 relative to Block 1, but AVGs showed the largest increase (see Figure 4A for a visualization). Thus, this result is partially in line with our hypotheses since we expected inter-individual differences in alpha oscillatory activity between video gamers and control participants and intra-individual differences between experimental blocks, but we did not expect the effects to interact with each other. None of the other main or interaction effects for medium or upper alpha ratios reached significance (see Tables 2, 3 for a more detailed inspection of the ANOVAs for medium or upper alpha ratios, respectively).

**TABLE 2:** ANOVA with GROUP [non-video gamers (NVGs) vs. non-action video gamers (NAVGs) vs. action video gamers (AVGs)] as between-subjects factor, TIME ON TASK (Block 1 vs. Block 2) and EXPOSURE DURATION (17.8, 35.6, 53.4, 89, 144.6, and 198 ms) as within-subject factors and medium alpha ratios (10–12 Hz) as dependent variable.

**Predictor**	***df*_Num_**	***df*_Den_**	***Epsilon***	***F***	***p***	**$\eta_{\text{G}}^{2}$**	***BF*_10_**
Group	2.00	35.00		0.47	0.626	0.02	
Time on task	1.00	35.00		17.65	0.000	0.02	>100
Group x Time on task	2.00	35.00		4.80	0.014	0.01	>100
Exposure duration	3.72	130.37	0.74	16.80	0.000	0.05	>100
Group × Exposure duration	7.45	130.37	0.74	0.90	0.510	0.01	
Time on task × Exposure duration	4.14	144.77	0.83	0.61	0.662	0.00	
Group × Time on task × Exposure duration	8.27	144.77	0.83	0.64	0.749	0.00	

*df_*Num*_ indicates degrees of freedom numerator. df_*Den*_ indicates degrees of freedom denominator. Epsilon indicates Greenhouse–Geisser multiplier for degrees of freedom, *p*-values and degrees of freedom in the table incorporate this correction. $\eta_{G}^{2}$ indicates generalized eta-squared. *BF*_10_ indicates Bayes factors in favor of the H_1_. *BF*_10_ was only reported for significant main or interaction effects. Raw medium alpha ratios were log-transformed using log_10_ to prepare them for statistical analyses based on linear models.*

**TABLE 3:** ANOVA with GROUP [non-video gamers (NVGs) vs. non-action video gamers (NAVGs) vs. action video gamers (AVGs)] as between-subjects factor, TIME ON TASK (Block 1 vs. Block 2) and EXPOSURE DURATION (17.8, 35.6, 53.4, 89, 144.6, and 198 ms) as within-subject factors and upper alpha ratios (12–14 Hz) as dependent variable.

**Predictor**	***df*_Num_**	***df*_Den_**	***Epsilon***	***F***	***p***	**$\eta_{\text{G}}^{2}$**	***BF*_10_**
Group	2.00	35.00		2.69	0.082	0.11	
Time on task	1.00	35.00		2.27	0.141	0.00	
Group × Time on task	2.00	35.00		0.67	0.516	0.00	
Exposure duration	3.63	127.05	0.73	5.41	0.001	0.02	>100
Group × Exposure duration	7.26	127.05	0.73	0.96	0.467	0.01	
Time on task × Exposure duration	4.59	160.63	0.92	0.77	0.564	0.00	
Group × Time on task × Exposure duration	9.18	160.63	0.92	1.17	0.315	0.00	

*df_*Num*_ indicates degrees of freedom numerator. df_*Den*_ indicates degrees of freedom denominator. Epsilon indicates Greenhouse–Geisser multiplier for degrees of freedom, *p*-values and degrees of freedom in the table incorporate this correction. $\eta_{G}^{2}$ indicates generalized eta-squared. *BF*_10_ indicates Bayes factors in favor of the H_1_. *BF*_10_ was only reported for significant main or interaction effects. Raw upper alpha ratios were log-transformed using log_10_ to prepare them for statistical analyses based on linear models.*

On top of that, the ANOVA with GROUP (NVGs vs. NAVGs vs. AVGs) as between-subjects factor and TIME ON TASK (Block 1 vs. Block 2) as within-subject factor for C parameter values revealed a significant interaction GROUP × TIME ON TASK, *F*(2,35) = 3.99, *p* = 0.027, $\eta_{\text{G}}^{2}$ = 0.05, *BF*_10_ = 0.22. This indicates that there were differential intra-individual differences in C parameter values between experimental blocks between video gamers and control participants. In detail, NVGs showed larger C values than video gamers in Block 1. In contrast, video gamers exhibited larger C values than NVGs in Block 2 with AVGs showing the largest increase (see Figure 4B). Thus, partially in line with our hypothesis, the temporal dynamics of TVA C parameter values were nicely paralleled by medium alpha ratios in video gamers but not in NVGs. None of the other main or interaction effects for C or K parameter values reached significance (for a detailed description of the ANOVAs for TVA C and K parameter values see Tables 4, 5, respectively).

**TABLE 4:** ANOVA with GROUP [non-video gamers (NVGs) vs. non-action video gamers (NAVGs) vs. action video gamers (AVGs)] as between-subjects factor, TIME ON TASK (Block 1 vs. Block 2) as within-subject factor and theory of visual attention speed of information processing parameter values (C parameter values) as dependent variable.

**Predictor**	***df*_Num_**	***df*_Den_**	***F***	***p***	**$\eta_{\text{G}}^{2}$**	***BF*_10_**
Group	2	35	0.33	0.720	0.01	
Time on task	1	35	0.67	0.418	0.00	
Group × Time on task	2	35	3.99	0.027	0.05	0.22

*df_*Num*_ indicates degrees of freedom numerator. df_*Den*_ indicates degrees of freedom denominator. $\eta_{G}^{2}$ indicates generalized eta-squared. *BF*_10_ indicates Bayes factors in favor of the H_1_. BF_10_ was only reported for significant main or interaction effects. Note that the low *BF*_10_ value for the GROUP × TIME ON TASK interaction was to be expected as neither the main effect GROUP nor the main effect TIME ON TASK were significant.*

**TABLE 5:** ANOVA with GROUP [non-video gamers (NVGs) vs. non-action video gamers (NAVGs) vs. action video gamers (AVGs)] as between-subjects factor, TIME ON TASK (Block 1 vs. Block 2) as within-subject factor and theory of visual attention visual short-term memory capacity parameter values (K parameter values) as dependent variable.

**Predictor**	***df*_Num_**	***df*_Den_**	***F***	***p***	**$\eta_{\text{G}}^{2}$**
Group	2	35	0.94	0.400	0.04
Time on task	1	35	0.16	0.694	0.00
Group × Time on task	2	35	1.82	0.178	0.01

*df_*Num*_ indicates degrees of freedom numerator. df_*Den*_ indicates degrees of freedom denominator. $\eta_{G}^{2}$ indicates generalized eta-squared.*

At last, we analyzed whether there was an association between the interaction effect for C and the interaction effect for medium alpha ratios by means of a regression analysis with C parameter difference values as criterion variable, medium alpha ratio difference values as predictor variable and GROUP as dummy variable (NVGs vs. NAVGs vs. AVGs) where NVGs were used as reference. One non-video gamer and one action video gamer had to be excluded from the analysis because they were identified as outliers. The regression analysis indicated that the model was significant, *R*^2^ = 0.41, *F*(5,30) = 4.10, *p* = 0.006, *BF*_10_ = 8.29. The estimated linear models for NVGs, NAVGs and AVGs can be inferred from equation (6), (7), and (8), respectively, where *Y* indicates C parameter difference values, *X* medium alpha ratio difference values, *i* the index corresponding to a participant and ε_i_ the residual term:

(6) $$N\,V\,G\,s:Y_{i}=-1.64-34.22*X_{i}+\varepsilon_{i}$$

(7) $$N\,A\,V\,G\,s:Y_{i}=2.76-6.47*X_{i}+\varepsilon_{i}$$

(8) $$A\,V\,G\,s:Y_{i}=-4.94+162.74*X_{i}+\varepsilon_{i}$$

**FIGURE 5:**
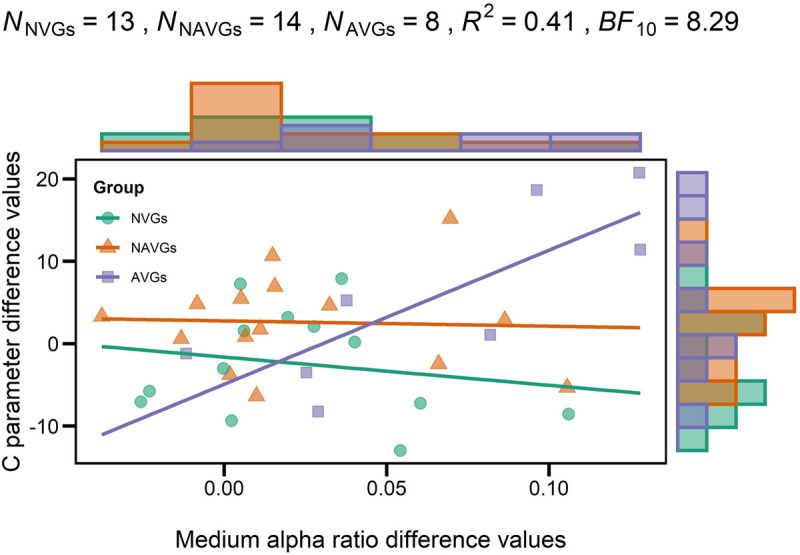
Regression model with each participant’s C parameter difference value as criterion variable, each participant’s medium alpha ratio difference value as predictor variable and GROUP [non-video gamers (NVGs) vs. non-action video gamers (NAVGs) vs. action video gamers (AVGs)] as dummy variable where NVGs were used as reference. Values > 0 indicate that participants’ values in the second experimental block were larger than those in the first block; values < 0 indicate that participants’ values in the first experimental block were larger than those in the second block. *N*_j_ indicates the number of participants per group, *R*^2^ the determination coefficient of the model, *BF*_10_ the evidence in support of the model fit. Group affiliation is indicated by different colors (NVGs: green, NAVGs: orange, AVGs: purple) and shapes (NVGs: circle, NAVGs: triangle, AVGs: square). Histograms opposite to the *y*- and *x*-axes indicate the distribution of C parameter and medium alpha ratio difference values for each group (NVGs: green, NAVGs: orange, AVGs: purple), respectively. Note that one non-video gamer and one action video gamer had to be excluded from the analysis as they were identified as outliers using Cook’s distance. While there were relatively small and negative relations between C parameter and medium alpha ratio difference values in NVGs and NAVGs, there was a strong positive association in AVGs, indicating that the association between intra-individual differences in both average medium alpha ratios and C parameter values between experimental blocks was particularly strong in AVGs.

By comparing the slope values between equations (6), (7), and (8), two differences between NVGs and NAVGs on the one side, and AVGs on the other side are observable: firstly, in NVGs and NAVGs there seems to be a negative relation between C parameter difference values and medium alpha ratio difference values, indicating that if NAVGs and NVGs showed an increase in alpha power attenuation in a frequency band from 10 to 12 Hz in the second experimental block relative to the first one, their speed of information processing capacity might decrease. In contrast, in AVGs, there was a positive association, suggesting that if AVGs showed such an increase, their speed of information processing might increase. Secondly, the sizes of the slope values in NVGs and NAVGs were relatively smaller than those of AVGs. For a visualization of the linear models of each group, see Figure 5. This was in line with our previous results for NVGs and AVGs but not NAVGs – though one should not pay too much attention to the slope value of the NAVGs’ model as it was quite small and hence not significant. Thus, our regression analysis seems to support our previous observation that intra-individual differences in alpha power attenuation (10–12 Hz) and C parameter values between experimental blocks may be differentially associated depending on gaming behavior; and judging by the size of the slope value, this seems to apply especially to AVGs. However, these results should be considered with caution as the slope estimates in the AVGs model were heavily biased by a rather small sample of participants. For a more detailed description of the regression analysis, see Table 6.

**TABLE 6:** Regression analysis with C parameter difference values as criterion variable, medium alpha ratio difference values as predictor variable and GROUP as dummy variable [non-video gamers (NVGs) vs. non-action video gamers (NAVGs) vs. action video gamers (AVGs)] where NVGs were used as reference.

**Predictor**	***b***	***b* 95% CI [LL, UL]**	**Fit**
Intercept	−1.64	[−6.09, 2.81]	
Medium alpha ratio difference values	−34.22	[−139.63, 71.18]	
NAVGs	4.40	[−1.62, 10.41]	
AVGs	−3.30	[−12.28, 5.68]	
Medium alpha ratio difference values × NAVGs	27.76	[−109.95, 165.47]	
Medium alpha ratio difference values × AVGs	196.96	[53.72, 340.20]	
			*R^2^* = 0.41 95% CI[0.05, 0.53] F(5, 30) = 4.10, p = 0.006, BF_10_ = 8.29

*b represents unstandardized regression weights. *LL* and *UL* indicate the lower and upper limits of a confidence interval without corrections for multiple comparisons, respectively. *R*^2^ indicates the determination coefficient. *BF*_10_ indicates the Bayes factor in favor of the model fit.*

## Discussion

Given that EEG alpha power modulations represent a neural substrate of attentional control, our results may support the idea that the conjunction between reward-related learning and attentional control represents a core mechanism in cognitive improvement in video gamers (Bavelier and Green, 2019). This is because, we found that inter-individual differences in speed of information processing as operationalized by TVA between video gamers and NVGs were associated with inter-individual differences in post-stimulus EEG alpha power attenuation from 10 to 12 Hz. Hereby, it was particularly interesting that video gamers did not outperform NVGs right from the start but in the course of performing the paradigm and that this was paralleled by an increase in alpha ERD in video gamers but not in NVGs – which is well in line with the learning to learn approach (Bavelier et al., 2012b; Green and Bavelier, 2012). Moreover, it was quite interesting that our regression analysis indicated that foremost AVGs might benefit from this increase in alpha ERD as they showed the strongest positive relation between TVA C parameter and medium alpha ratio difference values – which supports the idea that gamers might benefit specifically from playing action video games (Achtman et al., 2008). Therefore, our data suggest that attentional control as operationalized by EEG alpha power modulations might play a considerable role in learning mechanisms relevant for cognitive improvement in video gamers (Bavelier and Green, 2019).

We show that alpha ERD in a frequency range from 10 to 12 Hz may represent a neural substrate of speed of information processing as operationalized by TVA. Considering that the extent of ERD in a similar alpha frequency band after stimulus processing had already been related to reaction times (Nenert et al., 2012), this result was not surprising, but it might play a considerable role for new vistas on TVA. This is because, according to TVA (Bundesen, 1990; Kyllingsbaek, 2006; Dyrholm et al., 2011; Bundesen et al., 2015), visual information is processed by means of two core attentional processing components, i.e., filtering and pigeonholing, where filtering allows the selection of specific visual features, e.g., colors, while pigeonholing allows for the selection of visual categories, e.g., letters (Broadbent, 1970). Based on the assumption that all visual information is processed in parallel and that there is a limited capacity of the visual short-term memory store, filtering and pigeonholing represent mechanisms to determine the processing rate or probability for a visual stimulus to be encoded into the visual short-term memory store as described by the rate equation (9)

(9) $$v_{x}\,\left(i\right)=\eta \,\left(x,i\right)\,\beta_{i}\,\frac{w_{x}}{\sum_{z\in S}w_{z}}$$

where *v*_*x*_(*i*) indicates the processing rate that *x* is an element of category *i*, η(*x*,*i*) the sensory evidence that *x* is an element of *i*, β_*i*_ the perceptual decision bias to favor category *i* over other categories, and *w*_*x*_ the relative attentional weight in favor of an object *x* – which is divided by the sum of attentional weights of all remaining objects in the visual field *S*. The attentional weight term *w*_*x*_ can be described further by equation (10)

(10) $$w_{x}=\sum_{j\in G}\eta \,\left(x,j\right)\,\pi_{j}$$

where *j* indicates a visual category aka visual feature, *G* the set of all features that may be associated with a pertinence value, η(*x*,*j*) the sensory evidence that *x* belongs to feature *j*, and π_*j*_ the pertinence or weight in favor of a feature *j* (Dyrholm et al., 2011). Thus, β_*i*_ and *w*_*x*_ represent pigeonholing and filtering, respectively. On top of that, based on the assumption that the rate equation is dependent on the exposure duration of visual stimuli and the number of visual stimuli in the visual field, and that the attentional capacities of healthy humans are equally spread across the visual field, *v*_*x*_(*i*) may also be considered as a fraction of the total processing capacity at a given time point in a multi-stimulus setting where the processing capacity is spread across the whole visual field. Thus, the processing rate of a stimulus can also be described according to equation (11)

(11) $$v_{x}=C\,\frac{w_{x}}{\sum_{z\in S}w_{z}}$$

where *v*_*x*_ indicates the processing rate of an object *x*, *C* the fixed limited processing capacity measured in Hz or the number of elements that can be encoded per second (aka TVA’s speed of information processing parameter) and *w*_*x*_ the relative attentional weight in favor of object *x* (Dyrholm et al., 2011). Thus, according to TVA, visual speed of information processing is moderated by the attentional weighting term – or by an individual’s filtering capacity.

Based on Klimesch (1997, 2012) and Klimesch et al. (2011), one might expect that oscillatory activity in the lower alpha frequency band was associated with TVA’s rate equation. But in contrast, our data suggest that higher-level cognitive processing might be a mechanism either of the C parameter or the conjunction between the C and the attentional weight parameters as C parameter values were associated with alpha power attenuation in a frequency band from 10 to 12 Hz after stimulus processing. A potential explanation here might be that the two parameters may represent the retrieval of specific *knowledge systems* to semantically encode sensory information (Klimesch et al., 2011; Klimesch, 2012). According to Klimesch et al. (2011) and Klimesch (2012), a knowledge system refers to a neural network that is associated with implicit and explicit aspects of long-term memory. Hereby, alpha phase alignments in the medium or upper alpha frequency bands are assumed to represent the onset of retrieving from a knowledge system, while alpha power modulations in the medium or upper alpha frequency band may modulate the amplification of the retrieval of relevant and the suppression of the retrieval of irrelevant information from a knowledge system (Klimesch et al., 2011; Klimesch, 2012). But further research investigating the role of lower alpha oscillations and the conjunction between lower and medium or upper alpha oscillations and TVA C as well as *w*_*x*_ parameter values would be necessary to gain more insights on the role of knowledge systems in TVA.

Furthermore, we would like to discuss potential implications of our results at the intersection of computer sciences, health care and the gaming industry. Considering that there are already customized video games that are used for clinical purposes (Kollins et al., 2020); and video games where on-screen characters may even be controlled by means of brain oscillatory activity or event-related potentials using brain-computer interfaces (Bayliss, 2003; Nijholt et al., 2009), which may enable people with a physical disability to enjoy video gaming and feel more inclusive (see, Bos et al., 2010, for a more detailed review), one might question the significance of gaming effects associated with commercially available video games. One of the largest shortcomings of the latter is the lack of standardization. The issue here is that if we compare markers of neural activity and cognitive processing between participants who differ in their gaming behavior, or even if we compare changes in such markers associated with video gaming training regimes, we can hardly tell what differential characteristics between the groups or between the training regimes caused the inter-individual differences in neural activity and cognitive processing. On the other hand, research on gaming effects related to commercially available video games lays the foundation for the development of customized video games for clinical treatment, for fostering learning abilities, or for making commercially available video games even more entertaining. This is because, these frequent observations that inter-individual differences in neural and cognitive processing may be related to inter-individual differences in gaming behavior tell us that there may be aspects of commercially available video games that play a considerable role for human learning. Understanding the mechanisms underlying these phenomena, in turn, may then be the key for more significant or fruitful applications. For instance, if EEG alpha power modulations specifically in the medium and upper frequency bands turn out to be a robust neural substrate of cognitive improvements associated with playing commercially available video games, they could be used as a parameter for adjusting the complexity of the gameplay of video games in brain-computer interfaces.

With this being said, we would like to discuss some of the shortcomings in our experimental design as well. For instance, our individual group samples sizes were rather small which indicates a low statistical power. But considering that most of our statistical analyses (Pearson’s moment correlation tests, ANOVAs and regression analysis) were based on our total sample size (outliers not included), our statistical power should be acceptable. On top of that, we used type II sums of squares to compensate for unbalanced group samples (Langsrud, 2003); and in addition to that, our samples were similar to sample sizes of previous research on gaming effects (Li et al., 2009, 2011; Wu et al., 2012; Wilms et al., 2013; Schubert et al., 2015). Thus, we acknowledge that statistical power might be considered an issue in our study, but we argue that we used statistical methods that were less prone to individual group sample sizes and therefore, sufficient to identify reliable gaming effects. Furthermore, we did not find correlations between K parameter values and alpha ratios. We suggest that a reason for this may be that we analyzed a rather early time window where the CDA may not yet have been fully observable: Wiegand et al. (2014b), for instance, observed the onset of this event-related potential approximately 300 ms after stimulus processing and it persisted until a response was provided. By combining TVA data and EEG data from two different paradigms where no mask displays were used in the EEG paradigm but a blank screen of 900 ms, it was possible to investigate the CDA (Wiegand et al., 2014a,b, 2016). In contrast, based on our experimental design, it was more difficult to study this time window as we used mask displays and ratios based on 500 ms segments.

Besides, we did not exactly replicate previous findings where video gamers exhibited larger C parameter values than NVGs (Wilms et al., 2013; Schubert et al., 2015). We believe that one plausible reason for this might be that we used different classification criteria for participants than Wilms et al. (2013) and Schubert et al. (2015). Wilms et al. (2013), for instance, classified participants as experienced players if they had played action video games for more than 15 h a month, as casual players if they had played 4–8 h per month and as non-players if they played less than 2 h per months. In contrast, Schubert et al. (2015) classified participants as video game experts if they had played action video games at least 10 h a week in the last 6–12 months or as non-experts if they had played action video games less than 1 h a week in the last 6–12 months. In comparison, we also considered additional gaming habits to action video gaming, and we used different time constraints (Green et al., 2017). Thus, and in consideration that the classification of participants according to their gaming behavior may be in any case somehow arbitrary given that, for instance, action video games can be understood as video games where “under the most basic definition the player’s on-screen character can run, jump, roll, shoot, or fly, but the defining characteristic is that enemies and obstacles are overcome by physical means, rather than involved intellectual problem solving” (Next Generation, 1996, p. 29) (which is a rather unspecific description), we argue that differences in classification criteria are likely associated with slightly different gaming effects. Recruiting more specified and, hence, more differentiable groups might be a solution for this. For instance, Qiu et al. (2018) subdivided participants in AVGs and control participants based on their reported skill score in the video game League of Legends. Alternatively, in an attempt to increase effects between groups, a gaming group of professional e-sportsmen/sportswomen could be recruited.

Another limitation might be that despite our efforts to properly process the data, either our EEG power values, or our TVA estimates might not have been perfectly accurate because e.g., the factor exposure duration might have contaminated our procedures. Such contaminations are, unfortunately, inevitable using TVA-algorithms. Moreover, we need to point out that our data only show a correlational and not a causal relation between faster visual information processing and EEG alpha power modulations in gamers. To show a causal relation, one might need to apply an experimental design comprising a video gaming training regime.

Nevertheless, our data indicate that there may be inter-individual differences in event-related EEG alpha power modulations related to inter-individual differences in habitual gaming behavior, and that these modulations might go along with inter-individual differences in speed of visual information processing as operationalized by TVA. We conclude from this, that EEG alpha power modulations may be a promising neural substrate of alterations in visual cognitive processing in video gamers.

## Data Availability Statement

The raw data supporting the conclusions of this article will be made available by the authors, without undue reservation.

## Ethics Statement

The studies involving human participants were reviewed and approved by the local ethics review board. The patients/participants provided their written informed consent to participate in this study.

## Author Contributions

YH contributed to the conceptualization, data curation, formal analysis, investigation, methodology, project administration, and software development of this study. JM contributed significantly to the software development. JF contributed to the conceptualization and validation of this study. PS contributed to the conceptualization, funding acquisition, resources, and validation of this study. All the authors contributed to the article and approved the submitted version.

## Conflict of Interest

The authors declare that the research was conducted in the absence of any commercial or financial relationships that could be construed as a potential conflict of interest.

## Footnotes

1 https://wiki.python.org/moin/TkInter

2 https://www.spyder-ide.org/

3 https://real.kyiv.ua/avreal/download/#DLPORTIO_TABLE

4 https://docs.python.org/3.7/library/timeit.html

## References

[B1] Abdi H. (2010). “The greenhouse-geisser correction,” in *Encyclopedia of Research Design*, ed. Salkind N. (Thousand Oaks, CA: Sage), 544–548.

[B2] Achtman R. L. Green C. S. Bavelier D. (2008). Video games as a tool to train visual skills. *Restor. Neurol.Neurosci.* 26 435–446. 18997318 PMC2884279

[B3] Bavelier D. Achtman R. L. Mani M. Föcker J. (2012a). Neural bases of selective attention in action video game players. *Vis. Res.* 61 132–143. 10.1016/j.visres.2011.08.007 21864560 PMC3260403

[B4] Bavelier D. Green C. S. Pouget A. Schrater P. (2012b). Brain plasticity through the life span: learning to learn and action video games. *Annu. Rev. Neurosci.* 35 391–416. 10.1146/annurev-neuro-060909-152832 22715883

[B5] Bavelier D. Green C. S. (2019). Enhancing attentional control: lessons from action video games. *Neuron* 104 147–163. 10.1016/j.neuron.2019.09.031 31600511

[B6] Bayliss J. D. (2003). Use of the evoked potential P3 component for control in a virtual apartment. *IEEE Trans. Neural. Syst. Rehabil. Eng.* 11 113–116. 10.1109/TNSRE.2003.814438 12899249

[B7] Bediou B. Adams D. M. Mayer R. E. Tipton E. Green C. S. Bavelier D. (2018). Meta- analysis of action video game impact on perceptual, attentional, and cognitive skills. *Psychol. Bull.* 144 77–110. 10.1037/bul0000130 29172564

[B8] Bejjanki V. R. Zhang R. Li R. Pouget A. Green C. S. Lu Z.-L. (2014). Action video game play facilitates the development of better perceptual templates. *Proc. Natl. Acad. Sci. U.S.A.* 111 16961–16966. 10.1073/pnas.1417056111 25385590 PMC4250112

[B9] Benwell C. S. Y. London R. E. Tagliabue C. F. Veniero D. Gross J. Keitel C. (2019). Frequency and power of human alpha oscillations drift systematically with time-on- task. *NeuroImage* 192 101–114. 10.1016/j.neuroimage.2019.02.067 30844505 PMC6503153

[B10] Blacker K. J. Curby K. M. (2013). Enhanced visual short-term memory in action video game players. *Attent. Percept, Psychophys.* 75 1128–1136. 10.3758/s13414-013- 0487-0 23709068

[B11] Blacker K. J. Curby K. M. Klobusicky E. Chein J. M. (2014). Effects of action video game training on visual working memory. *J. Exp. Psychol. Hum. Percept. Perform.* 40 1992–2004. 10.1037/a0037556 25068696

[B12] Bland J. M. Altman D. G. (1995). Multiple significance tests: the Bonferroni method. *BMJ* 310:170. 10.1136/bmj.310.6973.170 7833759 PMC2548561

[B13] Boot W. R. Blakely D. P. Simons D. J. (2011). Do action video games improve perception and cognition? *Front. Psychol.* 2:226. 10.3389/fpsyg.2011.00226 21949513 PMC3171788

[B14] Bos D. P.-O. Reuderink B. van der Laar B. Gürkök H. Mühl C. Poel M. (2010). “Brain-computer interfacing and Games,” in *Brain-Computer Interfaces*, eds Tan D. S. Nijholt A. (London: Springer-Verlag), 149–178. 10.1007/978-1-84996-272-8_10

[B15] Broadbent D. E. (1970). “Stimulus set and response set: two kinds of selective attention,” in *Attention: Contemporary Theory and Analysis*, ed. Mostofsky D. (New York, NY: Appleton- Century Crofts), 51–60.

[B16] Bundesen C. (1990). A theory of visual attention. *Psychol. Rev.* 97 523–547. 10.1037/0033-295X.97.4.523 2247540

[B17] Bundesen C. Vangkilde S. Petersen A. (2015). Recent developments in a computational theory of visual attention (TVA). *Vis. Res.* 116 210–218. 10.1016/j.visres.2014.11.005 25458815

[B18] Cain M. S. Landau A. N. Shimamura A. P. (2012). Action video game experience reduces the cost of switching tasks. *Attent. Percept. Psychophys.* 74 641–647. 10.3758/s13414-012-0284-1 22415446

[B19] Cain M. S. Prinzmetal W. Shimamura A. P. Landau A. N. (2014). Improved control of exogenous attention in action video game players. *Front. Psychol.* 5:69. 10.3389/fpsyg.2014.00069 24575061 PMC3918660

[B20] Capotosto P. Babiloni C. Romani G. L. Corbetta M. (2009). Frontoparietal cortex controls spatial attention through modulation of anticipatory alpha rhythms. *J. Neurosci.* 29 5863–5872. 10.1523/JNEUROSCI.0539-09.2009 19420253 PMC2692025

[B21] Chisholm J. D. Kingstone A. (2012). Improved top-down control reduces oculomotor capture: the case of action video game players. *Atten. Percept. Psychophys.* 74 257–262. 10.3758/s13414-011-0253-0 22160821

[B22] Chun M. M. Golomb J. D. Turk-Browne N. B. (2011). A taxonomy of external and internal attention. *Annu. Rev. Psychol.* 62 73–101. 10.1146/annurev.psych.093008.100427 19575619

[B23] Colzato L. S. van Leeuwen P. J. A. van den Wildenberg W. P. M. Hommel B. (2010). DOOM’d to SWITCH: superior cognitive flexibility in players of first person shooter games. *Front. Psychol.* 1:8. 10.3389/fpsyg.2010.00008 21833191 PMC3153740

[B24] Cook R. D. (1977). Detection of influential observation in linear regression. *Technometrics* 19 15–18. 10.2307/1268249

[B25] Corbetta M. Patel G. Shulman G. L. (2008). The reorienting system of the human brain: from environment to theory of mind. *Neuron* 58 306–324. 10.1016/j.neuron.2008.04.017 18466742 PMC2441869

[B26] Deroy O. Spence C. Noppeney U. (2016). Metacognition in multisensory perception. *Trends Cogn. Sci.* 20 736–747. 10.1016/j.tics.2016.08.006 27612983

[B27] Dye M. W. G. Green C. S. Bavelier D. (2009). Increasing speed of processing with action video games. *Curr. Dir.Psychol. Sci.* 18 321–326. 10.1111/j.1467-8721.2009.01660.x 20485453 PMC2871325

[B28] Dyrholm M. Kyllingsbæk S. Espeseth T. Bundesen C. (2011). Generalizing parametric models by introducing trial-by-trial parameter variability: the case of TVA. *J. Math. Psychol.* 55 416–429. 10.1016/j.jmp.2011.08.005

[B29] Entertainment Software Association (2019). *2019 Essential Facts About the Computer and Video Game Industry.* Avaliable at: https://www.theesa.com/esa-research/2019-essential-facts-about-the-computer-and-video-game-industry/.

[B30] Finke K. Bublak P. Krummenacher J. Kyllingsbaek S. Muller H. J. Schneider W. X. (2005). Usability of a theory of visual attention (TVA) for parameter-based measurement of attention I: evidence from normal subjects. *J. Inter. Neuropsych. Soc.* 11 832–842. 10.1017/s1355617705050976 16519262

[B31] Föcker J. Cole D. Beer A. L. Bavelier D. (2018). Neural bases of enhanced attentional control: lessons from action video game players. *Brain Behav.* 8:e01019. 10.1002/brb3.1019 29920981 PMC6043695

[B32] Föcker J. Mortazavi M. Khoe W. Hillyard S. A. Bavelier D. (2019). Neural correlates of enhanced visual attentional control in action video game players: an event-related potential study. *J. Cogn. Neurosci.* 31 377–389. 10.1162/jocn_a_01230 29308981

[B33] Freunberger R. Klimesch W. Griesmayr B. Sauseng P. Gruber W. (2008). Alpha phase coupling reflects object recognition. *NeuroImage* 42 928–935. 10.1016/j.neuroimage.2008.05.020 18595738

[B34] Fries P. (2015). Rhythms for cognition: communication through coherence. *Neuron* 88 220–235. 10.1016/j.neuron.2015.09.034 26447583 PMC4605134

[B35] Gambacorta C. Nahum M. Vedamurthy I. Bayliss J. Jordan J. Bavelier D. (2018). An action video game for the treatment of amblyopia in children: a feasibility study. *Vis. Res.* 148 1–14. 10.1016/j.visres.2018.04.005 29709618 PMC5984723

[B36] Green C. S. Bavelier D. (2003). Action video game modifies visual selective attention. *Nature* 423 534–537. 10.1038/nature01647 12774121

[B37] Green C. S. Bavelier D. (2007). Action-video-game experience alters the spatial resolution of vision. *Psychol. Sci.* 18 88–94. 10.1111/j.1467-9280.2007.01853.x 17362383 PMC2896830

[B38] Green C. S. Bavelier D. (2012). Learning, attentional control, and action video games. *Curr.Biol.* 22 R197–R206. 10.1016/j.cub.2012.02.012 22440805 PMC3461277

[B39] Green C. S. Kattner F. Eichenbaum A. Bediou B. Adams D. M. Mayer R. E. (2017). Playing some video games but not others is related to cognitive abilities: a critique of Unsworth et al. (2015). *Psychol. Sci.* 28 679–682. 10.1177/0956797616644837 28346063

[B40] Green C. S. Pouget A. Bavelier D. (2010). Improved probabilistic inference as a general learning mechanism with action video games. *Curr. Biol.* 20 1573–1579. 10.1016/j.cub.2010.07.040 20833324 PMC2956114

[B41] Green C. S. Sugarman M. A. Medford K. Klobusicky E. Bavelier D. (2012). The effect of action video game experience on task-switching. *Comput. Hum. Behav.* 28 984–994. 10.1016/j.chb.2011.12.020 22393270 PMC3292256

[B42] Greenfield P. M. DeWinstanley P. Kilpatrick H. Kaye D. (1994). Action video games and informal education: effects on strategies for dividing visual attention. *J. Appl. Dev. Psychol.* 15 105–123. 10.1016/0193-3973(94)90008-6

[B43] Gruber W. R. Klimesch W. Sauseng P. Doppelmayr M. (2005). Alpha phase synchronization predicts P1 and N1 latency and amplitude size. *Cereb. Cortex* 15 371–377. 10.1093/cercor/bhh139 15749980

[B44] Hanslmayr S. Spitzer B. Bäuml K.-H. (2009). Brain oscillations dissociate between semantic and nonsemantic encoding of episodic memories. *Cereb. Cortex* 19 1631–1640. 10.1093/cercor/bhn197 19001457

[B45] Hazarika J. Dasgupta R. (2020). Neural correlates of action video game experience in a visuospatial working memory task. *Neural Comput. Appl.* 32 3431–3440. 10.1007/s00521-018-3713-9

[B46] Hazarika J. Kant P. Dasgupta R. Laskar S. H. (2018). Neural modulation in action video game players during inhibitory control function: an EEG study using discrete wavelet transform. *Biomed. Signal Process. Control* 45 144–150. 10.1016/j.bspc.2018.05.023

[B47] Jensen O. Mazaheri A. (2010). Shaping functional architecture by oscillatory alpha activity: gating by inhibition. *Front. Hum. Neurosci.* 4:186. 10.3389/fnhum.2010.00186 21119777 PMC2990626

[B48] Kassambara A. (2020). *rstatix: Pipe-Friendly Framework for Basic Statistical Tests. R Package Version 0.6.0.* Avaliable at: https://CRAN.R-project.org/package=rstatix (accessed June 18, 2020).

[B49] Klimesch W. (1997). EEG-alpha rhythms and memory processes. *Int. J. Psychophysiol.* 26 319–340. 10.1016/S0167-8760(97)00773-3 9203012

[B50] Klimesch W. (2012). α-band oscillations, attention, and controlled access to stored information. *Trends Cogn.Sci.* 16 606–617. 10.1016/j.tics.2012.10.007 23141428 PMC3507158

[B51] Klimesch W. Fellinger R. Freunberger R. (2011). Alpha oscillations and early stages of visual encoding. *Front. Psychol.* 2:118. 10.3389/fpsyg.2011.00118 21687470 PMC3108577

[B52] Klimesch W. Hanslmayr S. Sauseng P. Gruber W. R. Doppelmayr M. (2007a). P1 and traveling alpha waves: evidence for evoked oscillations. *J. Neurophysiol.* 97 1311–1318. 10.1152/jn.00876.2006 17167063

[B53] Klimesch W. Sauseng P. Hanslmayr S. (2007b). EEG alpha oscillations: the inhibition-timing hypothesis. *Brain Res. Rev.* 53 63–88. 10.1016/j.brainresrev.2006.06.003 16887192

[B54] Klimesch W. Schack B. Schabus M. Doppelmayr M. Gruber W. Sauseng P. (2004). Phase-locked alpha and theta oscillations generate the P1-N1 complex and are related to memory performance. *Brain Res. Cogn. Brain Res.* 19 302–316. 10.1016/j.cogbrainres.2003.11.016 15062867

[B55] Koepp M. J. Gunn R. N. Lawrence A. D. Cunningham V. J. Dagher A. Jones T. (1998). Evidence for striatal dopamine release during a video game. *Nature* 393 266–268. 10.1038/30498 9607763

[B56] Kollins S. H. DeLoss D. J. Cañadas E. Lutz J. Findling R. L. Keefe R. S. E. (2020). A novel digital intervention for actively reducing severity of paediatric ADHD (STARS-ADHD): a randomised controlled trial. *Lancet Digital Health* 2 e168–e178. 10.1016/S2589-7500(20)30017-0 33334505

[B57] Krishnan L. Kang A. Sperling G. Srinivasan R. (2013). Neural strategies for selective attention distinguish fast-action video game players. *Brain Topogr.* 26 83–97. 10.1007/s10548-012-0232-3 22614909 PMC3536985

[B58] Kühn S. Romanowski A. Schilling C. Lorenz R. Mörsen C. Seiferth N. (2011). The neural basis of video gaming. *Transl. Psychiatry* 1:e53. 10.1038/tp.2011.53 22833208 PMC3309473

[B59] Kyllingsbaek S. (2006). Modeling visual attention. *Behav. Res. Methods* 38 123–133. 10.3758/bf03192757 16817521

[B60] Langsrud Ø (2003). ANOVA for unbalanced data: use type ii instead of type iii sums of squares. *Stat. Comput.* 13 163–167. 10.1023/A:1023260610025

[B61] Lawrence M. A. (2016). *ez: Easy analysis and Visualization of Factorial Experiments. R Package Version 4.4-0.* Avaliable at: https://CRAN.R-project.org/package=ez (accessed November 02, 2016).

[B62] Levene H. (1960). “Robust tests for equality of variances,” in *Contributions to Probability and Statistics: Essays in Honor of Harold Hotelling*, eds Olkin I. Hotelling H. (Palo Alto, CA: Stanford University Press), 278–292.

[B63] Li R. Polat U. Makous W. Bavelier D. (2009). Enhancing the contrast sensitivity function through action video game training. *Nat. Neurosci.* 12 549–551. 10.1038/nn.2296 19330003 PMC2921999

[B64] Li R. Polat U. Scalzo F. Bavelier D. (2010). Reducing backward masking through action game training. *J. Vis.* 10:33 10.1167/10.14.33 21191129

[B65] Li R. W. Ngo C. Nguyen J. Levi D. M. (2011). Video-game play induces plasticity in the visual system of adults with amblyopia. *PLoS Biol.* 9:e1001135. 10.1371/journal.pbio.1001135 21912514 PMC3166159

[B66] Lüdecke D. Makowski D. Waggoner P. Patil I. (2020). *performance: Assessment of Regression Models Performance. R Package Version 0.4.7.* Avaliable at: https://CRAN.R-project.org/package=performance (accessed June 14, 2020).

[B67] McDermott A. F. Bavelier D. Green C. S. (2014). Memory abilities in action video game players. *Comput. Hum. Behav.* 34 69–78. 10.1016/j.chb.2014.01.018

[B68] Mishra J. Zinni M. Bavelier D. Hillyard S. A. (2011). Neural basis of superior performance of action videogame players in an attention-demanding task. *J. Neurosci.* 31 992–998. 10.1523/JNEUROSCI.4834-10.2011 21248123 PMC6632922

[B69] Moray R. D. Rouder J. N. (2018). *BayesFactor: Computation of Bayes Factors for Common Designs. R Package Version 0.9.12-4.2.* (accessed May, 19, 2018).

[B70] Nenert R. Viswanathan S. Dubuc D. M. Visscher K. M. (2012). Modulations of ongoing alpha oscillations predict successful short-term visual memory encoding. *Front. Hum. Neurosci.* 6:127. 10.3389/fnhum.2012.00127 22586390 PMC3347628

[B71] Neuwirth E. (2014). *RColorBrewer: ColorBrewer Palettes. R Package Version1.1-2.* Avaliable at: https://CRAN.R-project.org/package=RColorBrewer (accessed December 07, 2014).

[B72] Next Generation (1996). *The Next Generation Lexicon A To Z: A Definitive Guide To Gaming Terminology.* Avaliable at: https://archive.org/details/nextgen-issue-015/page/n29/mode/2up.

[B73] Nijholt A. Bos D. P. O. Reuderink B. (2009). Turning shortcomings into challenges: brain– computer interfaces for games. *Entertain. Comput.* 1 85–94. 10.1016/j.entcom.2009.09.007

[B74] Nobre A. C. van Ede F. (2018). Anticipated moments: temporal structure in attention. *Nat. Rev. Neurosci.* 19 34–48. 10.1038/nrn.2017.141 29213134

[B75] Olejnik S. Algina J. (2003). Generalized eta and omega squared statistics: measures of effect size for some common research designs. *Psychol. Methods* 8 434–447. 10.1037/1082-989X.8.4.434 14664681

[B76] Pavan A. Hobaek M. Blurton S. P. Contillo A. Ghin F. Greenlee M. W. (2019). Visual short-term memory for coherent motion in video game players: evidence from a memory- masking paradigm. *Sci. Rep.* 9:6027. 10.1038/s41598-019-42593-0 30988353 PMC6465596

[B77] Pfurtscheller G. Lopes da Silva F. H. (1999). Event-related EEG/MEG synchronization and desynchronization: basic principles. *Clin. Neurophysiol.* 110 1842–1857. 10.1016/S1388-2457(99)00141-8 10576479

[B78] Qiu N. Ma W. Fan X. Zhang Y. Li Y. Yan Y. (2018). Rapid improvement in visual selective attention related to action video gaming experience. *Front. Hum. Neurosci.* 12:47. 10.3389/fnhum.2018.00047 29487514 PMC5816940

[B79] R Core Team (2018). *R: A Language and Environment for Statistical Computing.* Vienna: R Foundation for Statistical Computing.

[B80] Rao R. P. N. (2005). Bayesian inference and attentional modulation in the visual cortex. *Neuroreport* 16 1843–1848. 10.1097/01.wnr.0000183900.92901.fc 16237339

[B81] Sauseng P. Klimesch W. Freunberger R. Pecherstorfer T. Hanslmayr S. Doppelmayr M. (2006). Relevance of EEG alpha and theta oscillations during task switching. *Exp. Brain Res.* 170 295–301. 10.1007/s00221-005-0211-y 16317574

[B82] Sauseng P. Klimesch W. Heise K. F. Gruber W. R. Holz E. Karim A. A. (2009). Brain oscillatory substrates of visual short-term memory capacity. *Curr. Biol.* 19 1846–1852. 10.1016/j.cub.2009.08.062 19913428

[B83] Schenk S. Lech R. K. Suchan B. (2017). Games people play: how video games improve probabilistic learning. *Behav. Brain Res.* 335 208–214. 10.1016/j.bbr.2017.08.027 28842270

[B84] Schmider E. Ziegler M. Danay E. Beyer L. Bühner M. (2010). Is it really robust? reinvestigating the robustness of ANOVA against violations of the normal distribution assumption. *Methodology* 6 147–151. 10.1027/1614-2241/a000016

[B85] Schubert T. Finke K. Redel P. Kluckow S. Müller H. Strobach T. (2015). Video game experience and its influence on visual attention parameters: an investigation using the framework of the theory of visual attention (TVA). *Acta Psychol.* 157 200–214. 10.1016/j.actpsy.2015.03.005 25834984

[B86] Schubert T. Strobach T. (2012). Video game experience and optimized executive control skills—On false positives and false negatives: reply to Boot and Simons (2012). *Acta Psychol.* 141 278–280. 10.1016/j.actpsy.2012.06.010

[B87] Shapiro S. S. Wilk M. B. (1965). An analysis of variance test for normality (Complete Samples). *Biometrika* 52 591–611. 10.2307/2333709

[B88] Spaak E. Fonken Y. Jensen O. de Lange F. P. (2016). The neural mechanisms of prediction in visual search. *Cereb. Cortex* 26 4327–4336. 10.1093/cercor/bhv210 26400919 PMC5066824

[B89] Standley D. (2018). *apaTables: Create American Psychological Association (APA) Style Tables. R package version 2.0.5.* Avaliable at: https://CRAN.R-project.org/package=apaTables (accessed August 29, 2018).

[B90] Strobach T. Frensch P. A. Schubert T. (2012). Video game practice optimizes executive control skills in dual-task and task switching situations. *Acta Psychol.* 140 13–24. 10.1016/j.actpsy.2012.02.001 22426427

[B91] Tanaka S. Ikeda H. Kasahara K. Kato R. Tsubomi H. Sugawara S. K. (2013). Larger right posterior parietal volume in action video game experts: a behavioral and voxel- based morphometry (VBM) study. *PLoS One* 8:e66998. 10.1371/journal.pone.0066998 23776706 PMC3679077

[B92] van Dijk H. van der Werf J. Mazaheri A. Medendorp W. P. Jensen O. (2010). Modulations in oscillatory activity with amplitude asymmetry can produce cognitively relevant event- related responses. *Proc. Natl. Acad. Sci. U.S.A.* 107 900–905. 10.1073/pnas.0908821107 20080773 PMC2818898

[B93] Vangkilde S. Bundesen C. Coull J. T. (2011). Prompt but inefficient: nicotine differentially modulates discrete components of attention. *Psychopharmacology* 218 667–680. 10.1007/s00213-011-2361-x 21629997 PMC3222829

[B94] Wickham H. (2016). *Ggplot2: Elegant Graphics for Data Analysis.* New York, NY: Springer-Verlag.

[B95] Wickham H. François R. Henry L. Müller K. (2019). *dplyr: A Grammar of Data Manipulation. R Package Version 1.0.0.* Avaliable at: https://CRAN.R-project.org/package=dplyr (accessed August 18, 2020).

[B96] Wiegand I. Hennig-Fast K. Kilian B. Müller H. J. Töllner T. Möller H.-J. (2016). EEG correlates of visual short-term memory as neuro-cognitive endophenotypes of ADHD. *Neuropsychologia* 85 91–99. 10.1016/j.neuropsychologia.2016.03.011 26972967

[B97] Wiegand I. Töllner T. Dyrholm M. Müller H. J. Bundesen C. Finke K. (2014a). Neural correlates of age-related decline and compensation in visual attention capacity. *Neurobiol. Aging* 35 2161–2173. 10.1016/j.neurobiolaging.2014.02.023 24684790

[B98] Wiegand I. Töllner T. Habekost T. Dyrholm M. Müller H. J. Finke K. (2014b). Distinct neural markers of TVA-based visual processing speed and short-term storage capacity parameters. *Cereb. Cortex* 24 1967–1978. 10.1093/cercor/bht071 23535180

[B99] Wilke C. O. (2019). *cowplot: Streamlined plot Theme and Plot Annotations for ‘ggplot2’. R Package Version 1.0.0.* Avaliable at: https://CRAN.R-project.org/package=cowplot (accessed September 08, 2020).

[B100] Wilms I. L. Petersen A. Vangkilde S. (2013). Intensive video gaming improves encoding speed to visual short-term memory in young male adults. *Acta Psychol.* 142 108–118. 10.1016/j.actpsy.2012.11.003 23261420

[B101] Worden M. S. Foxe J. J. Wang N. Simpson G. V. (2000). Anticipatory biasing of visuospatial attention indexed by retinotopically specific α-Bank electroencephalography increases over occipital cortex. *J. Neurosci.* 20 RC63–RC63. 10.1523/JNEUROSCI.20-06-j0002.2000 10704517 PMC6772495

[B102] Wu S. Cheng C. K. Feng J. D’Angelo L. Alain C. Spence I. (2012). Playing a first-person shooter video game induces neuroplastic change. *J. Cogn. Neurosci.* 24 1286–1293. 10.1162/jocn_a_00192 22264193

[B103] Wu S. Spence I. (2013). Playing shooter and driving videogames improves top-down guidance in visual search. *Attent. Percept. Psychophys.* 75 673–686. 10.3758/s13414-013-0440-2 23460295

